# The influence of exposure to *Toxoplasma Gondii* on host lipid metabolism

**DOI:** 10.1186/s12879-020-05138-9

**Published:** 2020-06-15

**Authors:** Fei Xu, Xiwan Lu, Ruitang Cheng, Yuwei Zhu, Sunhan Miao, Qinyi Huang, Yongliang Xu, Liying Qiu, Yonghua Zhou

**Affiliations:** 1 Department of Basic Medicine, Wuxi Medical School, Jiangnan University, Wuxi, Jiangsu 214122 P.R. China; 2 Key Laboratory of National Health Commission on Parasitic Disease Control and Prevention, Jiangsu Provincial Key Laboratory on Parasite and Vector Control, Jiangsu Institute of Parasitic Diseases and Public Health Research Center of Jiangnan University, Wuxi, 214064 Jiangsu China; 3 Wuxi Hospital of Traditional Chinese Medicine, Wuxi Affiliated Hospital of Nanjing Chinese Medicine University, Wuxi, Jiangsu 214071 P.R. China

**Keywords:** *Toxoplasma gondii*, Parasitic infection, Triglycerides, Total cholesterol, High-density lipoproteins, Low-density lipoproteins

## Abstract

**Background:**

Mounting evidence suggested a complex correlation between host lipid metabolism and *Toxoplasma gondii* (*T. gondii*) infection. However, the inherent association between *T. gondii* infection and host lipid state remains elusive either in mice or in human hosts.

**Methods:**

Through a study in a sample of 1045 healthy participants from eastern China, we determined the association of *T. gondii* infection and host lipid levels using serological methods. We then examined the host lipid levels in C57BL/6 J mice at both acute and chronic *T. gondii* infection stages (for a period up to 36 weeks post infection).

**Results:**

In our case-control study, *T. gondii* seropositive individuals had higher low-density lipoproteins (LDL) (*P* = 0.0043) and total cholesterol (TC) (*P* = 0.0134) levels compared to seronegative individuals. Furthermore, individuals with LDL (OR = 3.25; 95% CI:1.60–6.61) and TC (OR = 2.10; 95% CI:1.22–3.63) levels above the upper limit of normal range had higher odds ratio to be *T. gondii* IgG positive. Consistently, in vivo data revealed that a significantly increased LDL level was first observed at early acute stage but plateaued to later time (chronic infection with *T. gondii*).

**Conclusions:**

In both healthy population and *T. gondii*-infected mice, seropositive individuals had higher LDL level. Individuals with positive *T. gondii* IgG had more odds of being with LDL and TC abnormality. Latent *T. gondii* infection is common worldwide, potential medical interventions to host lipid metabolism may be a breakthrough point to the prevention and control of this parasite infection.

## Background

*Toxoplasma gondii* (*T. gondii*) is an obligate intracellular protozoan parasite. Currently, the estimated infection prevalence of *T.gondii* is relatively low in some countries, such as China (about 10%), UK (about 10%) and the USA (10 to 20%) [1–3]. It can be over 40% in South America and parts of Continental Europe [1]. *T. gondii* can infect almost all variety of warm-blooded animals. It invades and replicates in the intestinal system of hosts before entering the central nervous system. Mounting evidences show that *T. gondii* infection eventually triggers a wide range of clinical abnormalities, including miscarriage encephalitis [4, 5], Parkinson’s disease [6], schizophrenia [7], obsessive-compulsive disorder [8] and Alzheimer’s disease [9]. *T. gondii* transmits to humans mainly via the ingestion of food contaminated with cysts shed from bodily fluids or feces released by infected carriers or from undercooked meat [10–13]. Primary infection in healthy individuals is usually asymptomatic, or occasionally generates mild influenza-like symptoms, accompanied by self-limiting lymphadenopathy and hepatosplenomegaly [2, 14]. In immunosuppressed patients, *T. gondii* infection can lead to fatalities [15]. Given the prevalence of *T. gondii* in human beings, it is important to understand the parasite-induced physiological changes in mammals because of its potential far-reaching clinical consequences.

*T. gondii* has an obligate intracellular existence indicating its reliance on a source of essential nutrients that can be obtained from the intracellular medium. Normal replication within the parasitophorous vacuole (PV) [16] of *T. gondii* requires considerable amounts of lipids for membrane biogenesis. Although *T. gondii* has autonomous capacity to synthesize phospholipids [17, 18], it still needs to convert the host’s lipids intactly for membrane assembling due to the lack of the essential enzymes for sterols molecule synthesis [19]. A mobilization activity of host lipid resources to the parasites PV membrance (PVM) obviously exists in *T. gondii* [20, 21]. Owing to the profound effects of *T. gondii* on lipids in host, it is important to focus on the association between lipid levels and *T. gondii* infection in human subjects. However, studies about the *T. gondii* on the metabolism and uptake of lipids are not enough. So far only a few works have mentioned the association of *T. gondii* seropositivity with serum total cholesterol (TC) and high-density lipoprotein (HDL) levels in patients with schizophrenia [22].

Therefore, the correlation between the *T. gondii* seropositivity and serum lipid levels is poorly understood, especially in healthy individuals. A growing body of literatures have shown that lipid metabolic shifts occur in the host during acute *T. gondii* infection. However, the effect of these shifts has not been clarified in healthy population or in a chronic infection duration in vivo. This work is the first report revealing the association of *T. gondii* infection with serum TC, triglycerides (TG), low-density lipoprotein (LDL) and HDL levels, both in *T. gondii* infected mice model and a sample of healthy individuals.

## Methods

### Inclusion criteria of participants

We selected 1045 healthy participants from a physical examination population in Wuxi Affiliated Hospital of Nanjing Chinese Medicine University, Jiangsu Province, China. This study was performed from December 2018 to May 2019. Exclusion criteria for cases were as followed:1. Who didn’t accept to participate in the study; 2. Who had taken cardiovascular abnormality, pathoglycemia or dyslipidemia control drugs in the past 3 months. 3. Individuals with advanced *T. gondii*-related diseases; 4. Individuals with cardiac, renal, liver insufficiency or other obvious abnormal physical symptoms; 5. Individuals with severe mobility disorders or mental illness. The diagnosis of *Toxoplasma gondii* (*T. gondii*) infection in selected individuals met the criteria of the *T. gondii* control manual. The randomly selected healthy participants included 89 *T. gondii* seropositive individuals with 50 males and 39 females (average age of 31) and 956 *T. gondii* seronegative individuals with 532 males and 424 females (average age of 39). Occupation or socioeconomic status was not restrictive criteria for enrollment. Our study was in accordance with the standards of the 1964 Declaration of Helsinki (as revised in Brazil 2013), and with approval of Wuxi Affiliated Hospital of Nanjing Chinese Medicine University, China (NO. 2018011715).

### Infection of mice

A total of 120 female C57BL/6 J mice aged 6 weeks (SPF, weight median: 23.7 g, range: 21.4 to 25.5 g) in our experiments were purchased from the Comparative Medicine Center of Yangzhou University (experimental animal production license NO. SCXK-(su) 2012–0004). The mice were raised in the Animal Experiment Center of Jiangsu Institute of Parasite Prevention and Control (breeding environmental license number for experimental animals: SYXK-(su)2012–0034) and were housed in a humidity and temperature-controlled room on a light/dark cycle of 12 h, with free access to water and food. Mice were randomly divided into normal control and *T. gondii* infection group, with 60 mice in each group. Mice were orally infected with cysts suspension (including 6 to 8 cysts, isolated from the brains of mice infected with *T. gondii* two months post-infection) of *Toxoplasma gondii* Prugniaud (type II) strain. Mice in the normal control group were treated with normal saline by gavage. Five mice were dissected after anaesthetized by intraperitoneal injection of 1% sodium pentobarbital (50 mg/kg) at week 0, 1, 2, 3, 4, 5, 6, 7, 8, 16, 24 and 36 p.i.. The study was carried out after obtaining ethical clearance from Ethical Review Board (Ref. No. IACUC-JlPD-2,016,026) of Institute of Parasitic Disease Prevention and Control, Jiangsu, China.

### Lipids analysis

Total cholesterol (TC) (normal range: 2.9–5.68 mmol/L), high-density lipoprotein (HDL) (normal range: 0.9–2.19 mmol/L), low-density lipoprotein (LDL) (normal range: 1.9–3.6 mmol/L) and triglycerides (TG) (normal range: 0.34–1.92 mmol/L) of human serum samples were measured on the Beckman Kurt AU5800 automatic biochemical analyzer, according to the manufacturer’s recommendations. The range of normal lipid levels was set according to Guidelines for Prevention and Treatment of Dyslipidemia in Chinese Adults.

### Data analysis plan

Data were analyzed using SPSS 18.0 (SPSS Inc. Chicago, Illinois). Descriptive statistics included standard deviations and proportions for continuous variables, proportions for categorical variables. Means were compared by student t-test. The association of *T. gondii* seropositive and different lipid indexes was analyzed with the rank sum test. We calculated the sample with a reference seroprevalence of 8 to 22% [23] as the expected frequency of exposure in *T. gondii* seropositive groups. We calculated the odds ratio (OR) and 95% confidence interval (CI). Statistical significance was set at a *P* value < 0.05.

## Results

### T. Gondii seropositivity and serum lipid levels

One thousand forty-five healthy individuals (mean age = 47.29 ± 13.8 years old) were enrolled in this study. The rate of *T. gondii* seropositivity in the entire sample was 8.51% (89/956), which was within the range of the estimated infection rate in China and several other countries [23–25]. Demographics and clinical features of the participant samples by anti-*T. gondii* IgG status (positive or negative) are presented in Table 1. Male and female gender were basically evenly distributed in both *T. gondii* positive (male: 4.78%, female:3.73%) and negative groups (male:50.9%, female:40.6%) or in the entire sample (male: 55.7%, female:44.3%) (Table 1).

**Table 1:** Demographics of study samples

	T. gondii			*p* Value
	Seropositive	Seronegative	Combined	
	*N* = 89	*N* = 956	*N* = 1045	
	N (%)	N (%)	N (%)	
Sex				0.923
Male	50 (56.2)	532 (55.6)	582 (55.7)	
Female	39 (43.8)	424 (44.4)	463 (44.3)	
Age				0.794
< 40 years	28 (31.5)	284 (29.7)	312 (29.9)	
40 to < 50	21 (23.6)	246 (25.7)	267 (25.6)	
50 to < 60	26 (29.2)	246 (25.7)	272 (26.0)	
≥ 60	14 (15.7)	180 (18.8)	194 (18.6)	
TG				0.774
Low	0	0	0	
Normal	61 (68.5)	630 (65.9)	691 (66.1)	
High	28 (31.5)	326 (34.1)	354 (33.9)	
HDL				0.111
Low	2 (2.25)	45 (4.71)	47 (4.50)	
Normal	87 (97.8)	911 (95.3)	998 (95.5)	
High	0	0	0	
LDL				0.01
Low	2 (2.25)	40 (4.18)	42 (4.02)	
Normal	76 (85.4)	877 (91.7)	953 (91.2)	
High	11 (12.4)	39 (4.08)	50 (4.78)	
TC				0.024
Low	2 (2.25)	9 (0.94)	11 (1.05)	
Normal	68 (76.4)	836 (87.4)	904 (86.5)	
High	19 (21.3)	111 (11.6)	130 (12.4)	

Low: samples values exceed the lower limit of normal range;

High: samples values exceed the upper limit of normal range;

TG: triglycerides; HDL: high density lipoproteins;

LDL: low density lipoproteins; TC: total cholesterol

As demonstrated in Table 2, *T. gondii* seropositive individuals had nominally higher low-density lipoprotein (LDL) (*P* = 0.0043) and total cholesterol (TC) levels (*P* = 0.0134). Triglycerides (TG) and high-density lipoprotein (HDL) levels, as well as ratio of TG to HDL, LDL to HDL and TC to HDL did not differ between *T. gondii* seropositive and seronegative individuals, neither in all samples, nor in samples with abnormal serum lipid levels (Table 2). As indicated in Table 3, individuals with TC (*P* = 0.024) and LDL (*P* = 0.010) beyond normal range had significant higher chance of being seropositive compared to those with normal range of lipid levels. However, individuals who were positive for anti-*T. gondii* IgG had approximately twice the odds of being LDL (OR = 1.90; 95% CI:1.01–3.57; *P* < 0.05) and TC (OR = 2.15; 95% CI: 1.27-3.64; *P* < 0.05) abnormality compared to seronegative group (Table 3). It should be mentioned that, individuals with LDL (OR = 3.25; 95% CI:1.60–6.61; *P* < 0.05) levels above the upper limit of normal range had even higher odds ratio to be *T. gondii* IgG positive. In addition, there was no significant difference in risks for *T. gondii* infection among individuals with different sex and age (Table 3).

**Table 2:** Lipid levels of study samples in T.gondii seropositive and seronegative groups

	SPG (All) (N = 89)	SNG (All) (N = 956)	Values out of limits in SPG	Values out of limits in SNG	*P* value (1 vs. 2, 3 vs. 4)
TG (mmol/l)	1.754 ± 1.004	1.909 ± 1.637	2.870 ± 1.006(28)	3.234 ± 2.222(326)	0.3795, 0.3923
LDL (mmol/l)	2.795 ± 0.632	2.612 ± 0.573	3.885 ± 0.164(11)	4.081 ± 0.590(39)	0.0043, 0.2825
HDL (mmol/l)	1.230 ± 0.220	1.190 ± 0.211	–	–	0.0888, —
TC (mmol/l)	4.957 ± 1.021	4.714 ± 0.872	6.411 ± 0.529(19)	6.290 ± 0.652(111)	0.0134, 0.4432
TG/HDL (mmol/l)	1.534 ± 1.096	1.750 ± 1.896	–	–	0.2911, —
LDL/HDL (mmol/l)	2.338 ± 0.646	2.249 ± 0.576	–	–	0.1700, —
TC/HDL (mmol/l)	4.132 ± 1.047	4.060 ± 0.968	–	–	0.5074, —

SPG: *T.gondii* seropositive group; SNG: *T.gondii* seronegative group;

1 = *T.gondii* seropositive group (All); 2 = *T.gondii* seronegative group (All);

3 = Values out of limits in SPG; 4 = Values out of limits in SNG

All the values are formatted as: mean ± SD

**Table 3:** Odds ratios for T.gondii seropositive participants compared to seronegative

	T.gondii seropositive vs. seronegative	
	OR	95%CI
Sex		
Male	1	–
Female vs. Male	0.98	0.63, 1.52
Age		
< 40 years	1	–
40 to 50 vs. < 40	0.87	0.48, 1.56
50 to 60 vs. < 40	1.07	0.61, 1.88
≥ 60 vs. < 40	0.79	0.40, 1.54
TG		
Normal	1	–
Low vs. normal	–	–
High vs. normal	0.89	0.56, 1.41
HDL		
Normal	1	–
Low vs. normal	0.47	0.11, 1.95
High vs. normal	–	–
LDL		
Normal	1	–
Low vs. normal	0.58	0.14, 2.43
High vs. normal	3.25	1.60, 6.61
All vs. normal	1.9	1.01, 3.57
TC		
Normal	1	–
Low vs. normal	2.73	0.58, 12.9
High vs. normal	2.1	1.22, 3.63
All vs. normal	2.15	1.27, 3.64

Low: samples values exceed the lower limit of normal range;

High: samples values exceed the upper limit of normal range;

TG: triglycerides; HDL: high density lipoproteins;

LDL: low density lipoproteins; TC: total cholesterol

### Effect of T. gondii infection on weight and viscera of mice

**Fig. 1:**
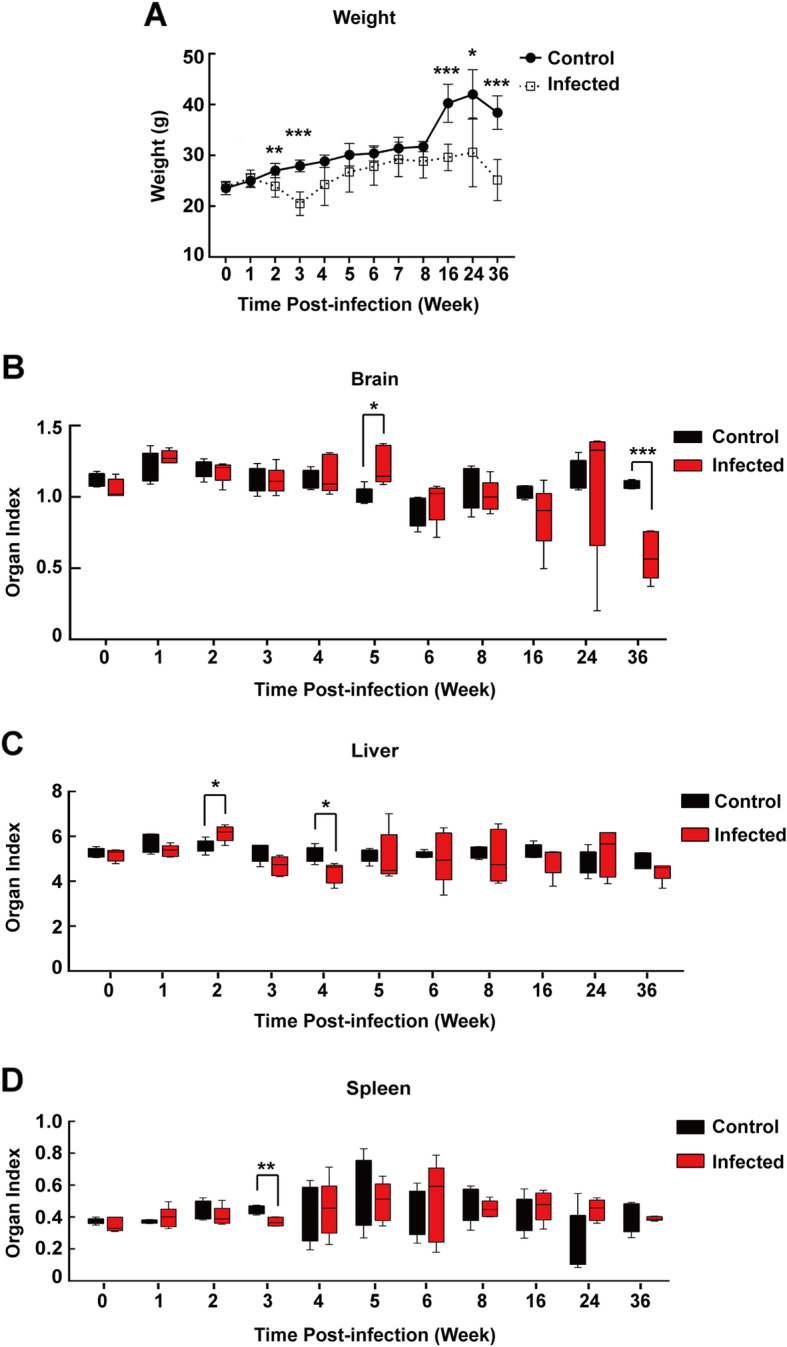
Weight curve and relative organ index in *T. gondii*-infected C57BL/6 J mice at certain time point post-infection (p.i.). **b** brain, **c** liver, **d** spleen index: organ weight (g) / body weight (g) (**a**). Values are expressed as mean ± S.D. (*n* = 5). *P < 0.05, **P < 0.01, ***P < 0.001 compared with the normal control group

To further verify the influences of *T. gondii* infection on the lipid status in vivo, healthy C57BL/6 J mice were orally infected with 6 to 8 cysts by gavage. Acute response post-infection (p.i.) promptly occurred during week 2 to week 3 post-infection (p.i.). The weight curve (*n* = 6) declined to the lowest point at week 3 p.i., which recovered gradually thereafter. Significant decrease was found again at week 16, 24, 36 p.i., possibly due to the deterioration of the host at late infection stage (Fig. 1a). In order to evaluate the effect of *T. gondii* infection on viscera, organ indexes were calculated. Cysts begin to form as early as one week after infection and persist in murine brain tissue for the rest of the host’s life. The diameter of cysts increased during the latent infection stage in brains, eventually causing slightly but significantly higher brain index in infected group at around week 5 p.i., as chronic stage began. Possibly due to the gradual severity of brain atrophy over time, a dramatically significant difference at week 36 p.i. occurred. *T. gondii* infection caused liver index rise to a certain extent at week 2 p.i. compared with control group (Fig. 1c), but conversely decreased at week 4 p.i.. Cysts transit to the tachyzoite stage within the digestive system and finally disseminate through the hosts' bloodstream to other tissues, once ingested by the host. Therefore, liver index increased at early stage post infection. Liver coefficient declined when cysts mainly metastasized to the brain, as approaching the chronic stage around week 5 p.i.. Perhaps due to immunosuppression at week 3 p.i., spleen index had a slight, albeit significant decrease in infected animals when compared to the control group (Fig. 1d).

### Kinetics of serum lipids in mice during T. gondii infection

**Fig. 2:**
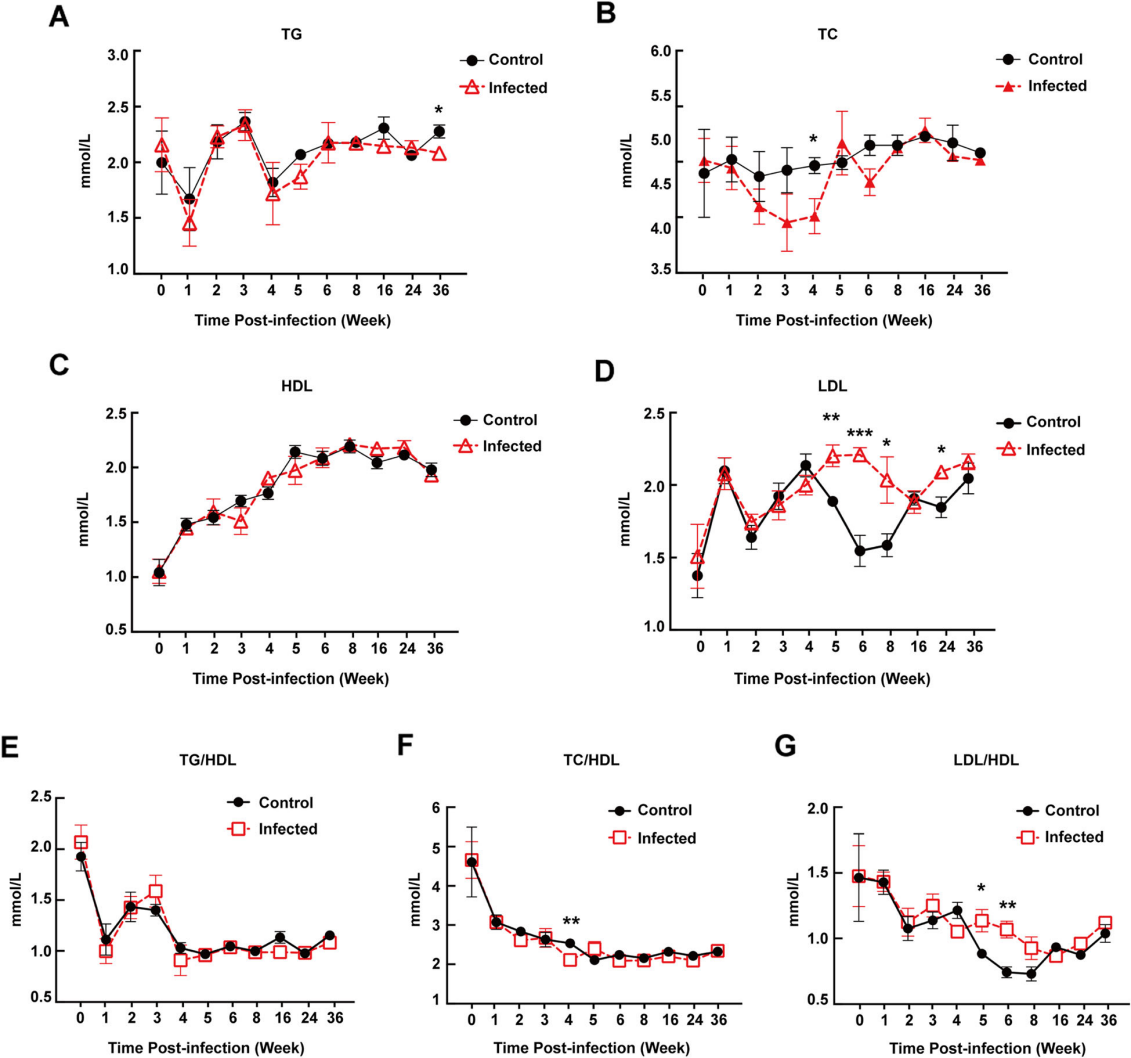
Kinetics of serum lipid levels (mean ± SD) during the course of *T. gondii* infection in C57BL/6 J mice. Groups of mice (*n* = 5) euthanized and bled at alternative weeks. **a** triglycerides (TG), **b** total cholesterol (TC), **c** high density lipoprotein (HDL), (D) low density lipoprotein (LDL) levels in infected and control groups. Asterisk: *P < 0.05, **P < 0.01, ***P < 0.001 *vs*. control mice

We conducted continuous lipid levels monitoring during 1 to 36 weeks after *T. gondii* infection. As presented in Fig. 2, no difference between control and infected group was seen before week 24 p.i.. However, at week 36 p.i., the mean value of TG level decreased slightly in infected group (Fig. 2a). No significant variation at any time point was observed in HDL level (Fig. 2c). The alteration of TG/HDL ratio was reflected in the same kinetics as HDL level (Fig. 2e), which was not comparable between control and infected groups. TC level of infected group decreased sharply from week 1 p.i. Although it increased thereafter, but a small, albeit significant (*P* = 0.045) difference remained at week 4 p.i. as compared to control groups (Fig. 2b). Almost the same kinetics was observed in TC/HDL ratio as TC level (Fig. 2f). It was worth mentioning that, LDL level of infected animals increased sharply from week 2 to 5 p.i., which showed a significantly difference at week 5 p.i. when compared with control groups as the chronic infection stage began. Although LDL level of infected group decreased thereafter from week 6 to week 16 p.i., a small but significantly increase occurred at week 24 p.i. (Fig. 2d). In Fig. 2g, LDL/HDL ratio in infected *vs*. control mice showed a trend similar to that in Fig. 2d: an increase occurred from week 5 to 6 p.i. but persisted comparable levels thereafter (Fig. 2g).

## Discussion

It has been shown that the cholesterol of *T. gondii* parasitophorous vacuole membrance (PVM) derived from endocytosed low-density lipoprotein (LDL) of the host, which was not endogenous synthesized by the host cells [19]. However, the exact mechanism underlying cholesterol uptake by intravacuolar *T. gondii* has not been completely elucidated. To determine the influences of *T. gondii* infection on the host lipid status in vivo, 1045 healthy samples were randomly selected and carefully excluded by our inclusion criteria to assess the serum lipid levels. We demonstrated that abnormal total cholesterol (TC) and LDL levels were positively correlated with *T. gondii* seropositivity. Individuals whose serum TC level fell outside the normal range or LDL level exceeded upper limit of normal range had higher odds ratio for *T. gondii* infection. Consistent with the above observations, individuals with *T. gondii* antibodies had higher serum LDL and TC levels compared to *T. gondii* seronegative samples. However, the mean value of LDL and TC levels exceeded upper limit of normal range had no significant difference within groups. Further studies are necessary to clarify how *T. gondii* mobilize the host’s lipid kinetics by a more systemic way. Thus, studies conducted in clinical samples rather than at cellular level are of critical importance.

As cholesterol in parasites is derived from LDL of the host, scavenging host lipids is critical for *T. gondii* survival [26]. Labeled fatty acid showed that *T. gondii* scavenge precursors from its host to synthesize full range of lipids [27]. However, a few studies about the association of *T. gondii* infection and host lipid metabolism showed obscure results [28–31]. Peer works reported that the only significant change in patients with schizophrenia was decreased serum triglyceride (TG) to high-density lipoprotein (HDL) ratio, observed in *T. gondii* seropositive group. Another study found no significant difference in TC level from cord blood serum between *T. gondii* seropositive and seronegative pregnant women [32]. Lipid metabolism in mice model with *T. gondii* infections is poorly understood. A following study showed that the major lipid metabolism alterations in mice included a decrease in serum HDL. TC level also decreased during acute phase (day 14 p.i.), which persisted until the end of the experiment (day 42 p.i.). An increase of LDL was observed at day 42 p.i., and no alterations in TG level were observed at any time point [33]. However, our results differed from the previous work, possibly due to the different mice (Swiss-Webster mice) or *T. gondii* strain (low virulence BGD-1 strain, human origin type-2) used in this work were not the same with ours. We demonstrated that the LDL level in infected animals increased as chronic phase began (week 5 to 6 p.i.). At this time point cysts formed and began to take advantage of the host-derived LDL. The increase of LDL was attributed to infection by *T. gondii*, as LDL is the major source from which *T. gondii* takes cholesterol [19]. To the contrary, serum TC levels decreased at day 7 p.i., at a time of established acute infection. Thus, we postulated that upon infection with *T. gondii*, uptake of cholesterol from serum LDL by rapidly multiplying tachyzoites triggered mobilization of cholesterol from the host’s liver to the peripheral blood in the form of VLDL and LDL. A generally decreasing trend was therefore observed in the TC curve of infected group, although it increased at later time points. It should be noted that, wild-type inbred strain of mice have low serum LDL levels unlike ApoE- or LDLr^−/−^ mice [34]. We postulated that was the reason why LDL levels lagged behind TC until the time of early chronic infection. Once LDL was mobilized to the periphery, it is utilized by rapidly proliferating tachyzoites, resulting in a constant LDL levels in the acute phase.

However, our studies had some limitations due to a relatively small cohort of subjects (1045 individuals) attending a local public hospital for physical examination. Moreover, this cross-sectional study did not allow the investigation on potential causality between host lipid metabolism and *T. gondii*, including a forward direction or vice versa. If abnormal lipid levels increased the risk of susceptibility to *T. gondii*? Or common causality, i.e., a shared factor causing both lipid abnormality and *T. gondii* susceptibility. Furthermore, we only demonstrated the physiological changes of host lipid levels after *T. gondii* infection but hadn’t clarified if such changes were due to the need for parasites proliferation or the response of hosts to infection.

Despite these limitations, to our knowledge, this is the first study to identify the association between host lipid metabolism and *T. gondii* infection among healthy individuals. Positive serum IgG and “not too high” OD values are usually indicated in chronic infection stage. The chronic infection stage of mice, generally 5 weeks after infection, is to some extent comparable with that of human beings. Our results showed that during chronic infection stage, the serum lipid levels of mice infected with *T. gondii* had similarity with that of human, especially the LDL phenotype. Moreover, we successively investigated lipid metabolism kinetics and monitored as long as 36 weeks p.i. in the wild-type murine host. We considered that the host’s lipid levels during acute or early chronic infection phase non-representative of the normal circumstances, since primary infection in healthy individuals was usually asymptomatic. In short, data presented here suggested that *T. gondii* had a potential effect on host lipid metabolism homeostasis during a long infection period. As host lipid metabolism is a complex process related to energy metabolism, immune system function etc., further studies are needed to clarify the mechanisms underlying this causality (i.e., whether abnormal lipid metabolism of the host to be the cause or effect to the risk of latent infection). Interventions targeting host lipid metabolism may be potential strategies for *T. gondii* infection since this parasite strictly rely on lipid metabolism of the host.

## Conclusion

In this study, we investigated the association between *T. gondii* seroprevalence and serum lipid levels in healthy population. Furthermore, experimental mice were used as acute and chronic infection model to verify the dynamic influence of *T. gondii* on serum lipid in vivo. All these data suggested that *T. gondii* infection affected lipid levels in the host. Interventions targeting host lipid metabolism may be potential strategies for *T. gondii* infection since this parasite strictly rely on lipid metabolism of the host.

## Data Availability

All the data supporting the study findings are within the manuscript. Additional detailed information will be shared upon request addressed to the corresponding author.

## Abbreviations

*T. gondii*: Toxoplasma gondii
p.i.: Post-infection
TG: Triglycerides
TC: Total cholesterol
LDL: Low density lipoproteins
HDL: High density lipoproteins
ACAT-1: Cholesterol acyltransferase 1
PV: Parasitophorous vacuole
OR: Odds ratio
CI: Confidence interval
